# Paroxysmal Nocturnal Hemoglobinuria Presenting With Progressive Biventricular Thrombi

**DOI:** 10.1155/crh/5349577

**Published:** 2026-06-03

**Authors:** Jordan S. Nunnelee, Garrett A. Weber, Nicholas P. Bergeron, Katherine E. Talbott, Andrew J. Layman, David Dingli, Richard C. Godby

**Affiliations:** ^1^ Division of Hematology, Department of Internal Medicine, Mayo Clinic, Rochester, Minnesota, USA, mayo.edu; ^2^ Department of Hematology and Oncology, Gundersen Medical Foundation, La Crosse, Wisconsin, USA, gundersenhealth.org; ^3^ Department of Internal Medicine, Mayo Clinic, Rochester, Minnesota, USA, mayo.edu; ^4^ Division of Cardiovascular Anesthesia, Department of Anesthesiology, Mayo Clinic, Rochester, Minnesota, USA, mayo.edu; ^5^ Department of Anatomic Pathology, Mayo Clinic, Rochester, Minnesota, USA, mayo.edu

## Abstract

A 34‐year‐old previously healthy woman presented with right‐sided, pleuritic chest pain three weeks after a viral respiratory infection. Labs revealed hemoglobin of 10.2 g/dL (nadir 7.0), platelets of 109 × 10^9^/L, LDH of 1129 U/L (Ref: 122‐222 U/L), undetectable haptoglobin, indirect hyperbilirubinemia, and elevated reticulocytes. Troponins were elevated. CRP was 184 mg/L (Ref: ≤ 5 mg/L). Peripheral smear was without schistocytes. Direct antiglobulin testing was negative. Urinalysis showed microscopic hematuria and positive hemosiderin.

Echocardiogram revealed normal left ventricular (LV) function with left lateral wall hypokinesis and a 2.5 × 3 cm LV thrombus. Cardiac MRI was concerning for myopericarditis and new right ventricular (RV) thrombus, later verified by biopsy. There was also an infarction of the RV endomyocardium. Anticoagulation was initiated. Bone marrow biopsy showed erythroid hyperplasia without clonal myeloid features. Flow cytometry showed 57% CD59 deficient red blood cells, 88% CD157 deficient monocytes, and 91% CD157 deficient neutrophils, consistent with a new diagnosis of paroxysmal nocturnal hemoglobinuria (PNH).

She was urgently vaccinated against encapsulated organisms, treated with eculizumab, and given antibacterial prophylaxis. Follow‐up imaging showed near resolution of thrombi; hemoglobin and hemolysis markers normalized.

PNH is a clonal hematopoietic disorder causing complement‐mediated hemolysis and thrombosis. While thrombosis typically affects atypical venous sites, intracardiac thrombi are rare. PNH was the patient’s only identified prothrombotic factor. This is the first reported case of intracardiac thrombosis in a previously healthy patient with new‐onset PNH. Early recognition and complement inhibition are critical to reduce hemolysis, thrombosis, and mortality in PNH.

## 1. Introduction

Paroxysmal nocturnal hemoglobinuria (PNH) is an acquired, clonal, somatic genetic disorder of hematopoietic stem cells, which leads to complement‐mediated intravascular hemolysis and an increased propensity for thrombosis and bone marrow failure [1]. PNH can occur at any age but typically presents in early‐to‐mid adulthood [2]. Thrombosis can occur in atypical sites and is the leading cause of mortality in patients with untreated PNH [1].

We describe a rare presentation of PNH in a young, otherwise healthy patient with progressive biventricular thrombi and concomitant myopericarditis.

## 2. Case Presentation

A 34‐year‐old previously healthy female presented to the emergency department with chest pain after a resolved viral upper respiratory infection (URI) three weeks prior. Her pain was right‐sided and worse with inspiration. She had no clinical signs or symptoms of heart failure.

An electrocardiogram (ECG) demonstrated nonspecific T‐wave abnormalities. Initial labs showed a hemoglobin of 10.2 g/dL, mean corpuscular volume of 99.1 fL (Ref: 78.2‐97.9 fL), leukocytes of 7.7 × 10^9^/L (3.4–9.6 × 10^9^/L), platelets of 109 × 10^9^/L (Ref: 157–371 × 10^9^/L), and a normal differential. High‐sensitivity Troponin T was 274 ng/L (Ref: ≤ 10 ng/L) on admission, 275 at 2 h, and 301 ng/L at 6 h. Peripheral smear did not reveal schistocytes, platelet clumping, or morphologic abnormalities of erythrocytes besides occasional macrocytosis. Her hemoglobin downtrended to a nadir of 7.0 g/dL. Lactate dehydrogenase (LDH) was 1129 U/L (Ref: 122‐222 U/L), haptoglobin was undetectable, total bilirubin was 1.9 mg/dL, and reticulocytes were persistently elevated with a peak absolute reticulocyte count of 200.6 x 10(9)/L. CRP was elevated to 184 mg/L (Ref: ≤ 5 mg/L). The direct antiglobulin test was negative. Urinalysis showed microscopic hematuria and hemosiderinuria. Creatinine and electrolytes were within normal limits.

**FIGURE 1:**
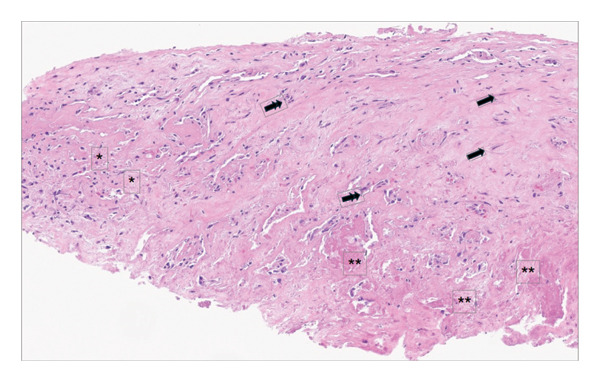
Gross pathology image demonstrating cardiac thrombus. Endomyocardial biopsy (hematoxylin and eosin, 200x original magnification): areas of collagen‐rich matrix with fibroblasts (arrow) and early neovascularization (double arrow) are in keeping with an organizing thrombus. Foci of scant cellular debris and loose stroma are suggestive of early degeneration (asterisk), whereas other regions show more eosinophilic fibrin‐rich areas with platelet accumulation, in keeping with more recent thrombosis (double asterisk).

**FIGURE 2:**
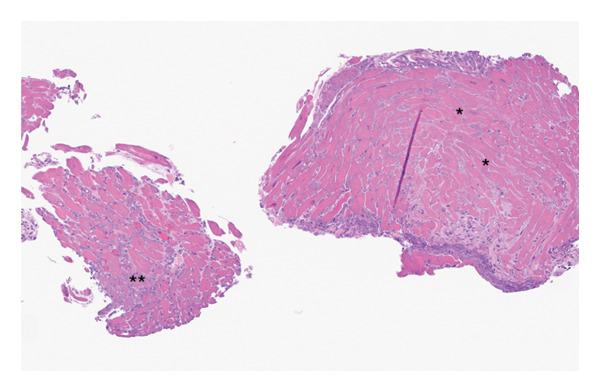
Gross pathology image demonstrating infarcted myocardium. Low‐power view of endomyocardial biopsy. Necrotic myocytes (asterisk) with necroinflammatory debris (double asterisk) and early fibrosis are present and consistent with acute to subacute myocardial infarction.

Transthoracic echocardiography (TTE) demonstrated normal left ventricle (LV) size and ejection fraction (EF) with LV wall motion abnormalities (hypokinesis in lateral segments of mid and apical ventricle) and a 2.5 × 3 cm intraventricular thrombus. There were no valvular abnormalities. Anticoagulation was initiated with a direct oral anticoagulant (DOAC). Cardiac magnetic resonance imaging (MRI) demonstrated findings concerning for patchy myopericarditis as well as LV thrombus and new right ventricle (RV) thrombus, subsequently confirmed via biopsy (Figure 1). There was no evidence of myocarditis seen histologically in the RV biopsy, but the biopsy demonstrated acute to subacute infarcted endomyocardium (Figure 2). There were also areas of mural thrombus adherent to infarcted myocardium. There was no definitive evidence of microvascular thrombi seen in the endomyocardium to explain the evidence of infarct. Cardiac catheterization showed normal coronary arteries. The CT chest angiogram was negative for pulmonary embolism. As there were no signs or symptoms of DVT, extremity Doppler ultrasounds were not performed. Abdominal ultrasound with Doppler was negative for splanchnic thrombosis. Serum testing for antiphospholipid antibodies and JAK2, CALR, and MPL was negative.

Bone marrow biopsy showed mildly hypercellular marrow with erythroid hyperplasia and no morphologic evidence of a clonal myeloid disorder. Antigen testing by flow cytometry showed 57% CD59 deficient red blood cells (RBCs), 23% demonstrated partial antigen loss (Type II PNH cells) and 34% demonstrated complete antigen loss (Type III PNH cells), 88% CD157 deficient monocytes, and 91% CD157 deficient neutrophils, consistent with a new diagnosis of PNH.

She was treated urgently inpatient with eculizumab and received vaccines against encapsulated organisms, including *Haemophilus influenzae* Type b (Hib), *Neisseria meningitidis* (MenACWY and MenB), and *Streptococcus pneumoniae* (PCV20), and given antibacterial prophylaxis with penicillin. While vaccination against Hib and pneumococcus is not required for terminal complement inhibitors, they are recommended for proximal pathway inhibitors [1]. Given an evolving clinical situation and possible changes in complement inhibitor therapy, she was vaccinated against all encapsulated organisms.

**FIGURE 3:**
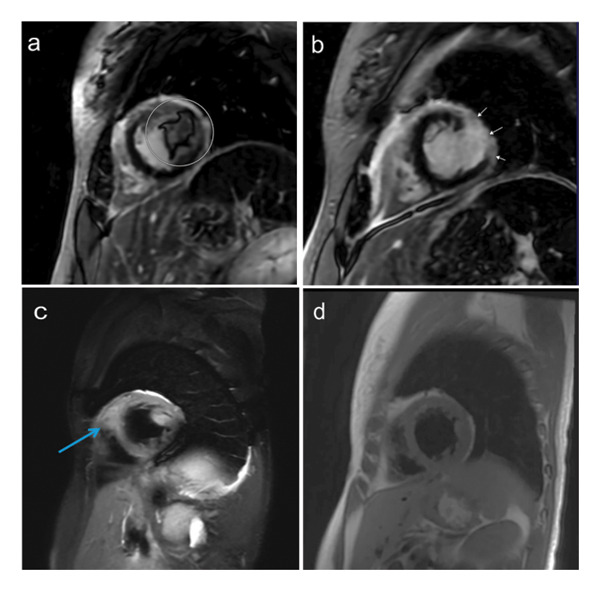
Cardiac delayed enhancement MRI imaging pretreatment (a and c) and 2.5 months posttreatment with anticoagulation and complement inhibition (b and d). LV thrombus seen in (a) (circle), with replacement of thrombus by mildly enhancing scar tissue laterally (arrows) in (b). RV thrombus seen in (c) with near‐resolution after 2.5 months of treatment (d).

Cardiac MRI 2.5 months after hospital discharge demonstrated near‐resolution of intraventricular thrombi (Figure 3). She transitioned to ravulizumab in the outpatient setting. Approximately three months from diagnosis and complement inhibitor initiation, hemoglobin increased to 13.0 g/dL, and hemolysis markers improved, including a normalized LDH to 198 u/L. Ferritin was 87 mcg/L, and she takes an iron‐containing prenatal vitamin.

## 3. Discussion

PNH is an acquired somatic genetic disorder of hematopoietic stem cells leading to loss‐of‐function mutations in the phosphatidylinositol glycan anchor biosynthesis Class A (PIGA) gene [1]. This causes decreased production of glycosylphosphatidylinositol (GPI) anchors that tether the naturally occurring complement inhibitors CD55 and CD59 to the surface of RBCs, leading to intravascular hemolysis and an increased propensity for thrombosis [2]. Thrombosis is the most significant cause of mortality in PNH [1].

The most common sites of thrombosis related to PNH are venous, predominantly intraabdominal including the hepatic, portal, and mesenteric veins [1, 2]. Intracardiac sites of thrombosis are extremely rare [3]. Myopericarditis can be associated with intraventricular thrombosis when there is persistently and severely reduced cardiac wall motion [4]. This is less consistent with this patient’s case. Her LV EF was normal to mildly reduced, with mild hypokinesis as opposed to akinesis of the lateral mid and apical wall. Her RV wall motion and EF were normal on multiple TTEs. Complicating the clinical picture is the right heart endomyocardial biopsy, which showed areas of infarct of unknown etiology.

A previously published case series on intraventricular thrombi demonstrated that thrombus formation within the heart is usually accompanied by a hypercoagulable state in the absence of atrial fibrillation or valvular disease [5]. Five out of seven cases of LV thrombus in the setting of normal ventricular function occurred in patients with a hypercoagulable state [5]. Our patient’s right heart function was normal, yet there was RV thrombus formation with evidence of endomyocardial infarct. The previously mentioned report does not provide data on right heart thrombus formation. We hypothesize that her new diagnosis of PNH was a driving etiology for thrombus development in the setting of her infarcted myocardium and MRI findings concerning myopericarditis. The etiology of these imaging findings is unclear and could be related to a preceding URI, local thrombus formation, endomyocardial infarct, or a combination thereof, without clear delineation of chronology given the acute presentation and clinical investigations. There is ongoing work to understand if there is an autoimmune process involved, in addition to the URI, that could have contributed to the myopericarditis.

This is a rare presentation of PNH due to the location of thrombi and concurrent cardiac findings with evidence of normal coronary arteries via catheterization and EKGs. PNH was the only identified risk factor for hypercoagulability. A previously published case report describes a young man with thymoma‐associated aplastic anemia presenting with a LV thrombus in the setting of subclinical PNH [6]. However, this is the first case to our knowledge demonstrating intracardiac thrombosis in a previously healthy person as the initial clinical presentation for PNH.

Complement inhibitors such as eculizumab inhibit the formation of the terminal membrane attack complex, thus inhibiting the mechanism of terminal complement‐induced hemolysis in PNH. Reduction of intravascular hemolysis leads to less release of free hemoglobin and other proinflammatory markers, reducing platelet activation, nitric oxide scavenging, and downstream thrombus formation [2]. Ultimately, with ongoing control of the PNH and thrombus resolution, the DOAC will be considered for discontinuation. She will require ongoing monitoring to assess for extravascular hemolysis, breakthrough hemolysis with complement‐amplifying conditions (e.g., pregnancy), and PNH evolution associated with bone marrow failure.

It is important to have a high index of suspicion for PNH in a patient presenting with hemolysis and thrombosis, as treatment is effective and distinct from other causes of hemolysis or thrombosis. Using targeted complement inhibitors to abrogate the acquired pathobiology reduces thrombosis and mortality.

## Funding

No funding was received for this study.

## Consent

All the patients allowed personal data processing, and informed consent was obtained from all individual participants included in the study.

## Conflicts of Interest

The authors declare no conflicts of interest.

## Data Availability

Research data are not shared.
